# To evaluate the efficacy and safety of different kinds of PDE5-Is with tamsulosin as a medical therapy for LUTS secondary to benign prostatic hyperplasia: A protocol for systematic review and meta analysis

**DOI:** 10.1097/MD.0000000000018712

**Published:** 2020-01-17

**Authors:** Chengquan Ma, Jianzhong Zhang, Zhonglin Cai, Hongjun Li

**Affiliations:** Department of Urology, Peking Union Medical College Hospital, Peking Union Medical College, Chinese Academy of Medical Sciences, Beijing, China.

**Keywords:** lower urinary tract symptoms, network meta-analysis, phosphodiesterase type 5 inhibitors, tamsulosin

## Abstract

**Background:**

Drug therapy for lower urinary tract symptoms (LUTS) secondary to benign prostate hyperplasia (BPH) is a major and popular method. However, the therapeutic strategy is still not clear enough up to now. The purpose of this study was to compare the relative safety and efficacy of different types of phosphodiesterase type 5 inhibitors (PDE5-Is) with tamsulosin for the treatment of LUTS secondary to BPH.

**Methods:**

Databases including PubMed, OpenGrey, Embase, Cochrane Library, and Web of Science will be searched to identify qualified studies. We will use the Stata version 13.0 to conduct the network meta-analysis (NMA) with a random or fixed effects model of Bayesian framework. International prostate symptom score (IPSS), maximum urinary flow fate (Qmax) and their credible intervals (CI) will be used to compare every medical intervention with the efficacy and safety, including sildenafil plus tamsulosin, tadalafil plus tamsulosin, vardenafil plus tamsulosin. And the ranking of probability of different interventions will be estimated by comparing the surface under the cumulative ranking curve (SUCRA).

**Results:**

A high quality-synthesis of the current evidence for comparing with different doses or types of PDE5-Is combined with tamsulosin to the treatment of LUTS secondary to BPH will be provided.

**Conclusions:**

This NMA and systematic review will generate evidence to help choose the best combination for treatment of LUTS secondary to BPH.

PROSPERO registration number: PROSPERO CRD 42019139062

## 1 Introduction

Both LUTS secondary to BPH and erectile dysfunction (ED) are common conditions in middle-aged or older men. Barbosa et al reported that 72.2% of men with LUTS were suffered from ED and demonstrated that LUTS was an independent risk factor for ED.^[1]^ The EAU guidelines for LUTS/BPH proposed α1-adrenoceptor antagonists (α1 blockers) as the first-line drug. PDE5-Is (sildenafil, tadalafil, vardenafil, udenafil, mirodenafil) as the first-line therapeutic drug for ED were also effective and safe in treating BPH/LUTS. Drug therapy for LUTS with or without ED is a major and popular method. However, the therapeutic strategy is still not clear enough up to now.

The first meta-analysis included five randomized controlled trials (RCTs) indicated that PDE5-Is can significantly improve the IPSS and IIEF compared with placebo though failed to find significant improvement in the Qmax.^[2]^ Subsequently, several meta-analyses have defined the efficacy and safety of PDE5-Is drugs alone or in combination with tamsulosin in LUTS/BPH with or without ED.^[3,4]^ The combination of 2 types of drugs has been found more effective and safe in alleviating LUTS than using each separately. All these articles aimed to assess the efficacy and the safety of combination therapy (tamsulosin and PDE5-Is) vs monotherapy (tamsulosin or PDE5-Is). However, there are no RCTs comparing safety and efficacy of different combined arms (such as sildenafil plus tamsulosin vs vardenafil plus tamsulosin vs tadalafil plus tamsulosin) to treat the LUTS secondary to BPH. Meta-analysis as a powerful tool can provide more reliable results by pooling the results of single studies. Therefore, we will conduct the network meta-analysis (NMA) through indirect or direct comparisons to find the best choice for improving IPSS and Qmax.

## 2 Methods

The protocol has been registered on the international prospective register of systematic reviews (PROSPERO registration number: CRD 42019139062) and was strictly reported based on the Preferred reporting items for systematic review and meta-analyses protocols statement. The study was approved by the Ethics Committee of Peking Union Medical College Hospital.

### 2.1 Data sources and extraction

A comprehensive literature electronic search will be performed to identify articles published before December 31, 2019 including PubMed, OpenGrey, Embase, Cochrane Library, and Web of Science. Only the English language articles will be reviewed and no limit about the publication date. We will use keywords as following: (PDE5-Is or sildenafil or tadalafil or vardenafil or udenafil or mirodenafil) and (LUTS) and (tamsulosin). Every reference listed in relevant studies will be checked to identify additional works not included in the electronic databases. Research papers selection will be performed by 2 independent reviewers (Chengquan Ma, Jianzhong Zhang). And the discrepancies will be discussed between reviewers or by a 3rd reviewer resolved.

RCTs will be included if they met the following criteria:

(1) The study compared sildenafil + tamsulosin and tamsulosin, tadalafil + tamsulosin and tamsulosin, vardenafil + tamsulosin and tamsulosin for the treatment of LUTS secondary to BPH.
(2) The study will provide endpoints for the clinical efficacy and safety of PDE5-Is with tamsulosin
(3) The primary endpoint is changes in symptoms and quality of life related to LUTS.

The exclusion criteria will be as follows:

(1) The study does not contain placebo groups or no-treatment groups or adequate data for inclusion
(2) The study is not the RCT types.
(3) The RCT not included IPSS or Qmax.

### 2.2 Quality assessment of the involved studies

The RCTs were evaluated by a 25-item Consolidated Standards of Reporting Trials checklist.^[5]^ The quality of the enrolled studies will be evaluated by Newcastle-Ottawa Scale star system (range, 0–9 stars), and the number of stars is positively associated with the quality of the study.

### 2.3 Data analysis for NMA

After extraction, all data will be pooled to perform a NMA. Outcomes of continuous variables will be expressed as mean difference (MD) with 95% CI for the IPSS and Qmax. NMA and forest-plot diagrams will be designed using a random-effect or fixed model. The hierarchy of competing interventions will be established using the SUCRA. The software will use the STATA version 13 (StataCorp LP, College Station, TX).

## 3 Discussion

BPH and associated LUTS is a progressive disease.^[6]^ Medical management of LUTS secondary to BPH with α1 blockers and/or 5α-reductase inhibitors is the first line treatment. However, the potential negative impact of these drugs may be the barrier for clinicians to prescribe these drugs for man. And especially the side effect on sexual function is of concern in young patients,^[7]^ though the research papers on their proof in this area were not in high quality and controversial. Recent research indicated that α1 blockers can impact upon sexual function and have been investigated about potential therapeutic for ED.^[8]^ Therefore, tamsulosin can be useful and safe to treat for LUTS secondary to BPH with or without ED. And the PDE5-Is is approved worldwide in therapeutic use for ED and was approved in the American for the treatment of signs and symptoms of BPH, and have since received regulatory approval in other countries for this indication. Then α1-adrenoceptor plus PDE5-Is may be perfect combination drugs for treat LUTS secondary to benign prostatic hyperplasia with or without ED.

PDE5-Is plus tamsulosin combination in treating man with LUTS secondary to BPH had been investigated. Kallidonis et al demonstrated that combination therapy (PDE5-I and α1 blockers) is more effective for the improvement of the IPSS, but less significant improvement for Qmax through a recent published meta-analysis.^[9]^ Our previous study has proven that the combination of PDE5-I plus a-blocker can significantly improve the efficacy of IPSS and Qmax for man with LUTS secondary to BPH, combination intervention way may be more favorable for these men.^[3]^ As we all know that PDE5-Is include five different drugs—sildenafil, tadalafil, vardenafil, udenafil, mirodenafil, however, there is no research to compare which regimen is the best choice to use for treating the LUTS secondary to BPH.

Several limitations of our NMA will be considered. The ethnicity may be diffident among the enrolled studies, which can increase the heterogeneity between studies and result in potential bias. Another limitation in this research may be that the treatment duration was different in RCTs, which could be affecting the outcomes of NMA.

## Author contributions

**Conceptualization:** Hongjun Li.

**Data curation:** Chengquan Ma.

**Formal analysis:** Chengquan Ma.

**Funding acquisition:** Hongjun Li.

**Investigation:** Hongjun Li.

**Methodology:** Chengquan Ma, Jianzhong Zhang.

**Project administration:** Hongjun Li.

**Software:** Chengquan Ma, Zhonglin Cai

**Supervision:** Hongjun Li.

**Writing – original draft:** Chengquan Ma.

**Writing – review and editing:** Hongjun Li.
